# Genetic variants in the SLC16A11 gene are associated with increased BMI and insulin levels in nondiabetic Chilean population

**DOI:** 10.20945/2359-3997000000359

**Published:** 2021-04-27

**Authors:** Lorena Mardones, Fanny Petermann-Rocha, Maria Adela Martinez-Sanguinetti, Ana Maria Leiva, Claudia Troncoso-Pantoja, Miquel Martorell, Nicole Lasserre, Natalia Ulloa, Francisco Perez-Bravo, Carlos Celis-Morales, Marcelo Villagran

**Affiliations:** 1 Universidad Católica de la Santísima Concepción Departamento de Ciencias Básicas Concepción Chile Departamento de Ciencias Básicas, Universidad Católica de la Santísima Concepción, Concepción, Chile; 2 University of Glasgow Institute of Cardiovascular and Medical Sciences BHF Glasgow Cardiovascular Research Centre Glasgow United Kingdom BHF Glasgow Cardiovascular Research Centre, Institute of Cardiovascular and Medical Sciences, University of Glasgow, Glasgow, United Kingdom; 3 University of Glasgow Institute of Health and Wellbeing Glasgow United Kingdom Institute of Health and Wellbeing, University of Glasgow, Glasgow, United Kingdom; 4 Universidad Austral de Chile Instituto de Farmacia Valdivia Chile Instituto de Farmacia, Universidad Austral de Chile, Valdivia, Chile; 5 Universidad Austral de Chile Instituto de Anatomía, Histología y Patología Valdivia Chile Instituto de Anatomía, Histología y Patología, Universidad Austral de Chile, Valdivia, Chile; 6 Universidad Católica de la Santísima Concepción Departamento de Salud Pública Centro de Investigación en Educación y Desarrollo Concepción Chile Centro de Investigación en Educación y Desarrollo, Departamento de Salud Pública, Universidad Católica de la Santísima Concepción, Concepción, Chile; 7 Universidad de Concepción Facultad de Farmacia Departamento de Nutrición y Dietética Concepción Chile Departamento de Nutrición y Dietética, Facultad de Farmacia, Universidad de Concepción, Concepción, Chile; 8 Universidad Santo Tomas Escuela de Nutrición y Dietética Sede los Ángeles Chile Escuela de Nutrición y Dietética, Universidad Santo Tomas, Sede los Ángeles, Chile; 9 Universidad de Concepción Facultad de Farmacia Departamento de Bioquímica Clínica e Inmunología Concepción Chile Centro de Vida Saludable, Departamento de Bioquímica Clínica e Inmunología, Facultad de Farmacia, Universidad de Concepción, Concepción, Chile; 10 Universidad de Chile Instituto de Nutrición y Tecnología de Alimentos (INTA) Santiago Chile Instituto de Nutrición y Tecnología de Alimentos (INTA), Universidad de Chile, Santiago, Chile; 11 University Católica del Maule Education, Physical Activity and Health Research Unit Human Performance Lab Talca Chile Human Performance Lab, Education, Physical Activity and Health Research Unit, University Católica del Maule, Talca, Chile

**Keywords:** *SLC16A11*, diabetes mellitus type 2, obesity, monocarboxylate transporter, hyperinsulinemia

## Abstract

**Objective:**

To study the association of SLC16A11 gene variants with obesity and metabolic markers in nondiabetic Chilean adults.

**Materials and methods:**

This cross-sectional study included 263 non-diabetic adults. The genotype of the rs75493593 polymorphism of SLC16A11 gene was performed by real-time PCR. It's association with adiposity markers (body weight, BMI, waist circumference and fat mass percentage), metabolic markers (glucose, insulin, HOMA_IR_, leptin, total cholesterol, LDLc, HDLc, triglycerides, ALT, GGT and hsCRP) and blood pressure was analyzed by linear regression.

**Results:**

The minor allele (T) of the SLC16A11 gene (rs75493593) has a frequency of 29.7% among Chileans. Risk genotypes (GT and TT) were associated with a significant 1.49 mU/l increase in plasmatic insulin for each copy of the minor allele (95% CI: 0.12, 2.87, p < 0.05). This association remained significant after adjusting for socio-demographic variables, physical activity and smoking (1.36 mU/l, 95% CI: 0.16, 2.58 p < 0.05), but was lost when BMI was included as a confounding factor. Higher BMI was also significantly associated with polymorphic genotypes in SLC16A11, independent of socio-demographic variables.

**Conclusion:**

The minor allele of the SLC16A11 gene (T) is highly prevalent among Chileans and is associated with increased insulin and BMI in nondiabetic individuals. These findings suggest that the genetic variant in SLC16A11 is not only associated with type 2 diabetes as previously shown in Mexicans, but is also related to early metabolic alterations in healthy subjects that may lead to type 2 diabetes.

## INTRODUCTION

Obesity has been identified as a major modifiable risk factor for type 2 diabetes (T2D). Pathophysiological conditions that occur with obesity, like low-grade inflammation, increased plasmatic-free fatty acids and insulin resistance, are directly related to the pathogenesis of T2D (1). As a reflection of the close interrelationship between these two conditions, it has been reported that more than 80% of people with T2D are overweight or obese (2). Furthermore, worldwide trends in the prevalence of T2D have closely mirrored those of obesity, doubling from 1980 to 2014 (3).

There are important differences in the prevalence of T2D among populations. Mexico and some Caribbean nations have over a 14.5% prevalence, which are the highest in the North American continent (4). In South America, Chile leads in T2D prevalence at 12.3%, according to the latest national health survey (5). Culturally-based lifestyle differences are a major contributor to the different prevalence of T2D among populations, including nutrition, physical activity and sedentarism. However, genetic variability related to ethnicity is also likely because the heritability of T2D and obesity has been estimated to be between 40% and 70% (6,7).

Genome-wide association studies (GWAS) for diabetes and obesity have been conducted mainly with European populations, revealing that both pathologies are highly polygenic and share some genetic determinants (6,7). For instance, the single nucleotide polymorphism (SNP) rs9939609 in the *FTO* gene has been identified as a common risk factor for obesity and T2D in several populations, including Chileans (8,9). Subsequent studies with non-European groups have discovered additional genetic variants with low prevalence among Europeans, but that are highly associated with T2D in other populations (10,11). For example, a haplotype of 5 SNPs in the *SLC16A11* gene was found in association with a 22% increase in T2D incidence in a Mexican population (12). Interestingly, this haplotype has a frequency of 50% in Mexican Native Americans but less than 1% in Europeans and Africans, therefore it was suggested that the haplotype may represent a common genetic T2D-susceptibility variant for Latin Americans (12). Although the association was later confirmed for Mexicans in the HCHS/SOL cohort, it was not replicated for other Latin American groups like Caribbeans, Central Americans or South Americans, even after the exclusion of young controls and adjustment for BMI (13). Subsequent in vitro studies have shown that the haplotype affects the aminoacidic sequence of the gene product, the monocarboxylate transporter type 11, which is most abundantly expressed in the thyroid gland and liver (14). In the latter tissue, these gene variants provoke reduced expression levels and impaired translocation of the transporter to plasma membrane, leading to intracellular accumulation of triglycerides (14).

Due to the high prevalence of obesity and T2D among Chileans and the heterogenic effect of the haplotype on T2D in different Hispanic groups, we studied the association of *SLC16A11* with adiposity and metabolic markers, using the rs75493593 SNP as a proxy for the 5 SNP haplotype in healthy Chilean adults.

## MATERIALS AND METHODS

The complete sample was composed of 472 individuals from the GENADIO study, but only 263 of them had information regarding the rs75493593 genotype in the *SLC16A11* gene. The GENADIO project was approved by the ethics committees of University of Concepcion, University of Chile and University of Glasgow; and took place between 2009 and 2011. The objective was to evaluate the prevalence of risk factors for cardiovascular diseases in Chile (15). The studied population included individuals of Mapuche and European descent living in the Biobío and Los Ríos regions. The Mapuche are the most populous indigenous group in Chile, accounting for a 79.8% of the indigenous people in the country (16). Individuals were selected who had no history of metabolic or cardiovascular disease or use of prescribed drugs (15).

### Allelic variant determination of *SLC16A11* gene

Allelic variants of the SNP rs75493593 in the *SLC16A11* gene were determined in genomic DNA isolated from blood leukocytes through QIAamp DNA Blood Midi Kit (QUIAGEN, Ltd, UK). Alleles were identified through real time PCR on an ABI 7900-HT thermocycler, using TaqMan pre-designed SNP genotyping assay with specific probes. All of the analyses were performed in duplicate, with a 98% of reproducibility.

### Adiposity markers

The anthropometric measurements were taken by trained personnel using standardized protocols (17). Body weight and height were determined with an electronic scale (TANITA TBF 300A, USA) and height rod (SECA A800, USA) with an accuracy of 100 g and 1 mm, respectively. Waist circumference (WC) and hip perimeter were measured with a non-distensible tape measure (SECA Model 201, US) using the anthropometric technique (17). Nutritional status was classified based on the World Health Organization's body mass index (BMI) cut-off points for adults: underweight: <18.5 kg/m^2^; normal weight: 18.5-24.9 kg/m^2^; overweight: 25.0-29.9 kg/m^2^ and obese: ≥30.0 kg/m^2^. The values used to define central obesity in men and women were WC ≥ 102 and 88 cm, respectively. Body composition was determined by measuring four skinfolds (bicipital, sub-scapular, supra-iliac and triceps) and the algorithm of Durnin and Womersley was applied to estimate the percentage of fat mass (18).

### Metabolic markers and blood pressure

Blood samples were obtained by venous puncture after 10 to 12 hours of fasting. Basal glycemia, total cholesterol (TC), HDL-cholesterol (HDLc) and triglycerides (TG) were measured using enzymatic end-point methods (Roche Diagnostics GmbH, Mannheim, Germany); and the enzymes gamma-glutamyltransferase (GGT) and alanine aminotransferase (ALT) were determined through kinetic assays (Randox Laboratories Ltd., Co. Antrim, Ireland). LDL-cholesterol (LDLc) was estimated using the Friedewald equation (19). Insulin and leptin were determined by ELISA (Diagnostic System Labs, TX, USA and Linco Research Inc., St. Louis MO, USA) and HOMA-IR (Homeostasis Model Assessment of Insulin Resistance) was determined through the following formula: insulinemia in fasting (mU/mL) x fasting glycemia (mg/dL)/405 (19). High sensitivity C-reactive protein (hsCPR) was measured by immunoturbidimetry (Kamiya Biomedical, Seattle, WA, USA). The average of two determinations was considered for each sample. Systolic (SBP) and diastolic (DBP) blood pressure were taken in supine position with an automatic tensiometer (OMRON M10-IT Healthcare UK Limited, Milton Keynes, UK) after a period of 10 minutes of rest.

### Sociodemographic and lifestyle variables

Sociodemographic data (age, gender, area of residence, educational level, income and ethnicity) and data associated with lifestyles were collected through validated surveys (15). Cardio-respiratory fitness was measured using the Chester Step Test and the results were registered in METs (Metabolic equivalents for task), according to Buckley and Cols. recommendations (20). Physical activity levels (PA) and sitting time were estimated by accelerometry of movement (Actigraph GTM1, USA). The intensity of PA and energy expenditure were determined by the Freedson algorithm (21).

### Statistical analysis

The characterization data of the studied population are presented as averages and standard deviations (SD) for continuous variables, and as a percentages for categorical variables. A linear regression analysis was applied to determine the association between rs75493593 polymorphism and adiposity makers (body weight, BMI, WC and fat mass %). The same analysis was applied to investigate the association with metabolic markers (glycemia, insulin, HOMA_IR_, TC, HDLc, LDLc, TG, ALT, GGT, hsCRP and leptin) and blood pressure (SBP and DBP).

The genotype of SNP rs75493593 was coded following an additive genetic model (0 = GG – homozygous for the protective allele, 1 = GT – heterozygous for the risk allele, 2 = TT – homozygous for the risk allele), and subsequently the increase in the health outcome (adiposity or metabolic marker) was estimated for each additional copy of the risk variant (T allele) by linear regression analysis. These results are presented as averages or beta coefficients along with their respective 95% confidence interval (95% CI).

The adiposity marker data were adjusted for confounding variables by using three statistical models: Model 0 – unadjusted; Model 1 – adjusted for age, gender, ethnicity, educational level, income, socioeconomic status and area of residence (urban/rural); Model 2 – adjusted for model 1 but also for PA, sitting time and smoking. An additional statistical model was included for the data on metabolic markers and blood pressure: Model 3, which incorporated BMI as a confounder. The distribution of the Hardy-Weinberg equilibrium of the alleles of the *SLC16A11* gene was evaluated by the Chi-square test. The STATA SE v14 program was used for all of the analyses. The level of significance was defined as p < 0.05.

## RESULTS

The cohort characteristics according to *SLC16A11* genotype (GG, TG or TT) are presented in Table 1. In general, carriers and non-carriers of risk allele (T) showed only minor differences in sociodemographic, physical activity and adiposity markers among (Table 1). However, the prevalence of the protective genotype (GG) is higher in Europeans than in Mapuches (65% versus 34%; Table 1). The allele frequency in the *SLC16A11* locus was 0.703 for the protective allele (G) and 0.297 for the risk allele (T), which is distributed according to the Hardy-Weinberg equilibrium (X^2^= 0.738, Table 2).

**Table 1:** Cohort characteristics according to *SLC16A11* genotype (rs75493593)

Variable		*SLC16A11* genotype (rs75493593)		
		GG	GT	TT
n		129	112	22
Age (years)		36.1 ± 13.8	37.8 ± 12.3	34.1 ± 11.9
Gender (% women)		57	58	50
Place of residency (% urban)		64	54	64
Ethnia (%)				
	European	65	55	54
	Mapuche	34	44	45
Education (%)				
	Elementary	12.4	22.5	13.6
	Secondary	53.4	32.4	72.7
	Higher	34.1	40.1	13.6
Income (%)				
	Low	28.9	34.5	31.8
	Medium	14.8	15.5	9.9
	High	56.2	50.0	59.1
Smoking (%)				
	Yes	58	50	59
	No	41	50	41
Physical activity & fitness				
	Physical activity (MET/min/week)	872.9 ± 287.9	912.6 ± 279.0	825.5 ± 329.7
	Sitting time (min/day)	525.3 ± 92.6	514.5 ± 87.7	555.4 ± 103.4
Adiposity				
	Body weight (kg)	70.2 ± 10.5	70.9 ± 10.6	72.5 ± 9.3
	BMI (kg/m^2^)	27.2 ± 3.8	27.9 ± 3.6	28.6 ± 3.9
Nutritional status (%)				
	Underweight	0.8	0	0
	Normal	25.6	25	18.2
	Overweight	46.5	50.9	45.4
	Obese	27.1	24.1	36.4
Waist circumference (cm)		94.7 ± 12.0	96.4 ± 9.9	98.4 ± 10.3
Central obesity (%)		59.7	56.2	63.6
Fat mass (%)		29.3 ± 4.6	29.3 ± 4.7	29.2 ± 4.6

Data presented as mean and standard deviation for continuous variables and as % for categorical variables.

**Table 2:** Allele frequency of rs75493593 in *SLC16A11* gene

rs75493593	n	Genotype frequency (%)	Allele frequency (%)	p value for HWE
GG	129	49.1	70.3	0.738
GT	112	42.6		
TT	22	8.4	29.7	

HWE: Hardy Weinberg Equilibrium.

**Figure 1:**
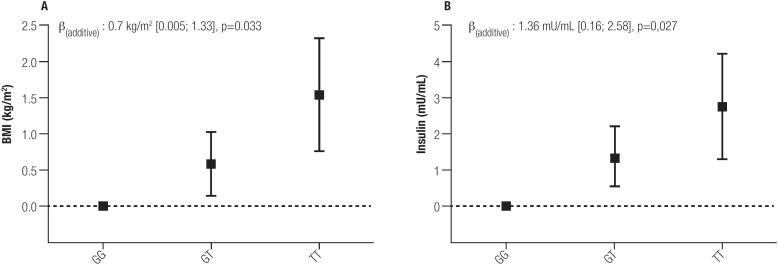
Association between *SLC16A11* genotype with BMI (A) and insulin (B). Data presented as differences between the reference allele (G) and the genotypes bearing the risk allele (T) and their respective standard errors. The analysis was adjusted by age, gender, ethnicity, educational level, income, place of residence, physical activity and smoking (Model 2).

The association between genotype in the *SLC16A11* locus and adiposity markers are presented in Table 3 and Figure 1A. Although we found higher body weight, BMI and waist circumference in carriers of the risk haplotype, only BMI showed a statistically significant increase for each copy of the risk allele. In the unadjusted model, BMI increased by 0.7 kg/m^2^ for each copy of the risk allele but the change was not significant (p = 0.052). When the association was adjusted by socio demographic variables in Model 1, it remained not significant (p = 0.053) and the increase in BMI was reduced to 0.65 kg/m^2^ for each copy of the risk allele. Only when physical activity variables were included in the fully adjusted model did the association reached significance (p = 0.033). The strength of the association remained practically unchanged in the model 3, with an increase of 0.7 kg/m^2^ (95% CI: 0.05; 1.33) in BMI for each copy of the risk allele.

**Table 3:** Association of *SLC16A11* genotype (rs75493593) with adiposity markers

Variables		*SLC16A11* genotype (rs75493593)			Effect of the additive genetic model	p value
		GG	GT	TT		
Body weight (kg)						
	Model 0	70.2 (68.4; 72.0)	70.9 (67.0; 72.9)	72.6 (68.2; 77.0)	1.00 (-0.99; 2.98)	0.324
	Model 1	70.3 (68.6; 72.0)	70.9 (69.1; 72.7)	72.4 (68.2; 76.6)	0.87 (-1.02; 2.77)	0.366
	Model 2	70.2 (68.5; 70.9)	71.0 (69.2; 72.9)	72.3 (68.1; 76.4)	0.96 (-0.91; 2.84)	0.312
BMI (kg/m^2^)						
	Model 0	27.2 (26.6; 27.9)	28.0 (27.2; 28.6)	28.6 (27.0; 30.2)	0.70 (-0.01; 1.40)	0.052
	Model 1	27.3 (26.7; 27.9)	27.8 (27.1; 28.4)	28.9 (27.4; 30.3)	0.65 (-0.01; 1.31)	0.053
	Model 2	27.3 (26.7; 27.9)	27.9 (27.2; 28.5)	28.8 (27.4; 30.2)	0.70 (0.05; 1.33)	0.033
Waist circumference (cm)						
	Model 0	94.7 (92.8; 96.6)	96.4 (94.4; 98.5)	98.4 (93.8; 103.0)	1.8 (-0.28; 3.89)	0.090
	Model 1	94.4 (93.0; 96.8)	96.1 (94.1; 98.1)	98.9 (94.3; 103.4)	1.63 (-0.43; 3.69)	0.122
	Model 2	94.8 (93.0; 96.7)	96.2 (94.2; 98.2)	98.7 (94.2; 103.3)	1.71 (-0.35; 3.76)	0.103
Fat mass (%)						
	Model 0	29.3 (28.5; 30.2)	29.4 (28.5; 30.2)	29.2 (27.2; 31.2)	-0.02 (-0.91; 0.86)	0.959
	Model 1	29.3 (28.6; 30.1)	29.4 (28.6; 30.2)	294 (27.6; 30.2)	0.02 (−0.80; −0.85)	0.952
	Model 2	29.3 (28.5; 30.0)	29.4 (28.6; 30.2)	29.3 (27.5; 31.1)	0.06 (−0.75; 0.88)	0.879

Data presented as means and their 95% CI. Analysis were adjusted as described in methods section. The effect of the additive genetic model represent the change in the outcome per 1 additional copy of the risk allele.

The association between genotype in the *SLC16A11* locus and metabolic markers are presented in Table 4 and Figure 1B. Among glucidic metabolic markers, we found a significant association between the risk allele of *SLC16A11* with higher insulin levels but not with glycemia, HOMA_IR_ or leptin levels. In the unadjusted model, the mean insulin levels was 1.49 mU/mL higher (95% CI: 0.12; 2.87; p = 0.034) for each copy of the risk allele. This association remained significant after adjusting for sociodemographic variables in Model 1 (p = 0.046) and for physical activity variables in Model 2 (p = 0.027). However, the association lost significance (p = 0.150) when we included BMI as a confounder in Model 3. Regarding lipidic markers, we did not find any significant association between the risk allele and changes in total cholesterol, HDLc, LDLc or triglyceride levels. Finally, no significant association with liver enzymes ALT or GGT, the inflammatory marker hsCPR or blood pressure was found in our population.

**Table 4:** Association of *SLC16A11* (rs75493593) with metabolic markers

Variables		*SLC16A11* genotype (rs75493593)			Effect of the additive genetic model	p value
		GG	GT	TT		
Glycemia (mg/dL)						
	Model 0	101.5 (98.9; 105.1)	96.9 (93.0; 100.7)	105.5 (96.9; 114.1)	−0.81 (−4.74; 3.13)	0.687
	Model 1	101.5 (98.2; 104.8)	96.7 (93.1; 100.3)	106.4 (98.4; 114.4)	−0.56 (−4.26; 3.13)	0.765
	Model 2	101.3 (98.0; 104.6)	97.0 (93.5; 100.6)	106.0 (98.1; 113.9)	−0.36 (−3.98; 3.24)	0.841
	Model 3	101.4 (98.1; 104.7)	97.0 (93.4; 100.5)	105.8 (97.8; 113.7)	−0.54 (−4.20; 3.11)	0.770
Insulin (mU/mL)						
	Model 0	6.0 (4.8; 7.3)	7.4 (6.1; 8.8)	9.2 (6.1; 12.2)	1.49 (0.12; 2.87)	0.034
	Model 1	6.2 (5.0; 7.3)	7.3 (6.0; 8.5)	9.0 (6.2; 11.8)	1.28 (0.02; 2.54)	0.046
	Model 2	6.1 (5.0; 7.2)	7.4 (6.2; 8.6)	8.8 (6.2; 11.5)	1.36 (0.16; 2.58)	0.027
	Model 3	6.4 (5.4; 7.4)	7.2 (6.1; 8.4)	8.0 (5.6; 10.5)	0.83 (−0.30; 1.96)	0.150
Leptin (ng/mL)						
	Model 0	13.0 (10.7; 15.3)	13.8 (11.2; 16.2)	11.6 (6.1; 17.2)	−0.83 (−2.62; 2.45)	0.949
	Model 1	13.0 (10.8; 15.3)	13.8 (11.3; 16.2)	11.5 (6.1; 16.8)	−0.17 (−2.62; 2.27)	0.891
	Model 2	13.0 (10.8; 15.2)	13.8 (11.4; 16.2)	11.4 (6.0; 16.8)	−0.14 (−2.59; 2.30)	0.909
	Model 3	13.5 (11.3; 15.6)	13.5 (11.2; 15.8)	10.2 (5.0; 15.4)	−0.95 (−3.33; 1.43)	0.435
HOMA_IR_						
	Model 0	1.59 (1.27; 1.92)	1.80 (1.45; 2.15)	2.23 (1.45; 3.01)	0.27 (−0.08; 0.63)	0.130
	Model 1	1.63 (1.34; 1.92)	1.76 (1.45; 2.08)	2.21 (1.51; 2.91)	0.22 (−0.10; 0.54)	0.173
	Model 2	1.60 (1.32; 1.88)	1.81 (1.50; 2.10)	2.16 (1.49; 2.82)	0.25 (−0.56; 0.55)	0.331
	Model 3	1.67 (1.42; 1.93)	1.76 (1.48; 2.04)	1.97 (1.34; 2.59)	0.12 (−0.17; 0.40)	0.418
TC (mg/dL)						
	Model 0	181.2 (172.8; 189.5)	180.6 (171.5; 189.6)	189.5 (169.4; 209.7)	−0.62 (12.92;11.68)	0.921
	Model 1	181.4 (173.4; 189.3)	179.7 (171.1; 188.3)	192.7 (171.5; 211.9)	2.63 (-6.11; 11.36)	0.554
	Model 2	180.8 (173.0; 188.5)	180.6 (172.1; 189.0)	191.7 (173.0; 210.5)	3.10 (-5.43; 11.63)	0.475
	Model 3	183.6 (177.7; 190.4)	178.8 (171.4; 186.2)	184.6 (168.0; 201.1)	-1.67 (-9.26; 5.91)	0.664
HDLc (mg/dL)						
	Model 0	36.1 (33.4; 38.7)	38.2 (35.3; 41.0)	31.4 (25.0; 37.8)	−0.46 (−3.38; 2.47)	0.758
	Model 1	36.2 (33.6; 38.8)	38.2 (35.4; 40.9)	30.8 (24.6; 36.9)	−0.73 (−3.56; 2.10	0.612
	Model 2	36.2 (33.7; 38.8)	38.0 (35.3; 40.8)	31.0 (24.8; 37.1)	−0.81 (−3.63; 2.00)	0.572
	Model 3	35.4 (33.1; 37.8)	38.6 (36.0; 41.1)	33.1 (27.5; 38.6)	−0.60 (−1.98; 3.18)	0.650
LDLc (mg/dL)						
	Model 0	122.8 (113.8; 131.8)	121.6 (111.9; 131.3)	135.9 (114.3; 157.6)	3.32 (-6.52; 13.16)	0.507
	Model 1	122.8 (114.2; 131.3)	120.9 (11.7; 130.2)	139.3 (118.7; 160.0)	4.08 (-5.33; 13.50)	0.394
	Model 2	122.2 (113.8; 130.6)	121.8 (112.7; 130.9)	138.3 (118.2; 158.6)	4.57 (-4.65; 13.78)	0.330
	Model 3	125.2 (117.8; 132.6)	119.8 (111.9; 127.8)	130.4 (112.7; 148.2)	−0.71 (−8.85; 7.44)	0.865
TG (mg/dL)						
	Model 0	112.6 (102.5; 122.8)	104.9 (94.0; 115.8)	111.8 (87.3; 136.2)	-3.51 (-14.59; 7.58)	0.536
	Model 1	113.2 (103.7; 122.6)	103.9 (93.7; 114.1)	113.7 (91.0; 136.5)	-3.68 (-14.04; 6.68)	0.485
	Model 2	112.8 (103.4; 122.1)	104.5 (94.4; 114.7)	113.0 (90.4; 135.6)	-3.32 (-13.60; 6.96)	0.525
	Model 3	115.4 (106.6; 124.1)	102.8 (93.4; 112.3)	106.2 (85.1; 127.2)	-7.89 (-17.52; 1.74)	0.108
ALT (U/L)						
	Model 0	37.0 (33.1; 41.0)	36.8 (32.5; 41.0)	40.1 (30.5; 49.7)	0.77 (-3.58; 5.12)	0.727
	Model 1	37.5 (33.6; 41.4)	30.1 (31.9; 40.3)	41.0 (31.6; 50.4)	0.44 (-3.82; 4.71)	0.837
	Model 2	37.2 (33.4; 41.0)	36.5 (32.4; 40.6)	40.5 (31.4; 49.6)	0.70 (-3.43; 4.84)	0.738
	Model 3	37.9 (34.3; 41.6)	36.0 (32.1; 40.0)	38.5 (29.7; 47.3)	−0.63 (−4.66; 3.39)	0.756
GGT (U/L)						
	Model 0	32.7 (27.8; 37.6)	33.3 (28.0; 38.6)	39.4 (27.5; 51.2)	2.18 (-3.20; 7.57)	0.426
	Model 1	33.1 (28.5; 37.7)	32.8 (27.8; 37.8)	39.2 (28.1; 50.3)	1.63 (-3.41; 6.68)	0.525
	Model 2	32.9 (28.4; 37.5)	33.1 (28.1; 38.0)	38.9 (27.8; 49.9)	1.79 (-3.22; 6.80)	0.482
	Model 3	33.9 (29.5; 38.3)	32.4 (27.7; 37.2)	36.3 (25.7; 46.9)	0.08 (-4.78; 4.94)	0.974
hsCRP (mg/L)						
	Model 0	1.41 (1.17; 1.64)	1.24 (0.98; 1.49)	1.31 (0.73; 1.88)	−1.01 (−0.36; 0.15)	0.441
	Model 1	1.40 (1.17; 1.62)	1.23 (0.99; 1.48)	1.38 (0.83; 1.93)	−0.07 (−0.32; 0.18)	0.559
	Model 2	1.39 (1.16; 1.61)	1.26 (1.01; 1.50)	1.36 (0.82; 1.90)	−0.06 (−0.31; 0.18)	0.618
	Model 3	1.46 (1.26; 1.66)	1.21 (0.99; 1.42)	1.16 (0.67; 1.63)	−0.20 (−0.42; 0.02)	0.081
SBP (mmHg)						
	Model 0	124.2 (121.3; 127.1)	121.2 (118.1; 124.3)	122.9 (115.8; 129.9)	-1.66 (-4.82; 1.51)	0.304
	Model 1	124.4 (121.8; 127.1)	120.8 (117.9; 123.6)	123.7 (117.2; 130.2)	-1.76 (-4.17; 1.19)	0.242
	Model 2	124.4 (121.7; 127.1)	120.8 (117.9; 130.2)	123.7 (117.2; 130.2)	-1.73 (-4.68; 1.23)	0.252
	Model 3	124.7 (122.0; 127.4)	120.6 (117.8; 129.5)	122.8 (116.3; 129.3)	-2.26 (-5.21; 0.69)	0.132
DBP (mmHg)						
	Model 0	75.8 (73.6; 78.0)	75.3 (72.9; 77.6)	75.9 (70.6; 81.2)	−0.23 (−2.64; 2.16)	0.844
	Model 1	6.0 (73.9; 78.2)	75.0 (72.6; 77.3)	76.5 (71.3; 81.7)	−0.30 (−2.66; 2.06)	0.803
	Model 2	75.9 (73.8; 70.0)	75.1 (72.8; 77.4)	76.3 (71.2; 81.5)	−0.21 (−2.56; 2.13)	0.857
	Model 3	76.1 (74.0; 78.3)	75.0 (72.7; 77.2)	75.6 (70.5; 80.8)	−0.63 (−2.97; 1.71)	0.597

Data presented as means and their 95% CI. Analysis were adjusted as described in methods section. The effect of the additive genetic model represent the change in the outcome per 1 additional copy of the risk allele.

## DISCUSSION

The GWAS developed by the SIGMA Consortium for the Mexican population first revealed the association of five exonic variants of the *SLC16A11* gene with the development of T2D (12). The five polymorphisms of this haplotype respectively generate a silent mutation (L187L) and four missense mutations in the gene product (V137I, D127G, G340S and P443T) (14). Interestingly, these five SNPs segregate together, enabling us to use the SNP rs75493593 corresponding to P443T, as a proxy for this haplotype (12). The prevalence of the 5-SNP haplotype has been estimated at 50% among Mexicans of indigenous origin, 28% among Mexicans of mixed indigenous-European descent, 12% in Asians and less than 2% in the European population (12,22). Interestingly, African populations present a different haplotype that encompasses only two of the 5 SNPs, specifically to D127G and L187L. This haplotype has a prevalence of 35% among Africans, but it is not associated with T2D (12). A recent study with the HCHS/SOL cohort confirmed the association of between the 5-SNP haplotype and T2D among Mexicans, but not among other Latin American groups like South Americans, Central Americans and Caribbeans (13). The lack of association in the latter populations could be related directly to their high African ancestry (23). However the lack of association among South American populations is puzzling because their genetic background and the haplotype prevalence are similar to those of Mexican mestizos. Although our study did not investigate the association between the haplotype and T2D, our data show higher insulin levels among Chilean haplotype carriers, and therefore supports an association with T2D among South Americans.

Mapuches are the main ancestral population in the central and southern regions of Chile and account for 79.8% of the total indigenous people in the country (16). An estimated 44% of Chileans have an Amerindian genetic component, which is similar to the proportion found in Mexican demographics (24). The 29.7% prevalence of the *SLC16A11* risk haplotype among Chileans that we report, is consistent with the similar genetic background of Mexicans. Despite our small sample size, we can observe that the prevalence of the risk haplotype is more prevalent among people of Mapuche descent than among those of Hispanic descent. Previous studies have shown that rural Mapuches have a very low prevalence of T2D (4.1%), while the prevalence is twice as high among urban Mapuches (25). In addition, Mapuches present a high prevalence of particular SNPs that are not shared with other indigenous populations in the Chilean territory, but these previous studies did not analyzed the *SLC16A11* locus (26). Given to the strong effect of rural/urban environments on T2D risk of among Mapuches it would be interesting to investigate whether the association between *SLC16A11* genetic variants and T2D interacts with environmental factors like place of residency, educational level or variables related to physical activity.

Our study reveals an association between the risk haplotype of the *SLC16A11* gene and higher levels of BMI and insulin among Chilean nondiabetic population. The higher insulin in carriers of the risk haplotype does not represent a true hyperinsulinemic state (e.g. > 15 mIU/L) (27); rather, it is related directly to the nutritional state because the association was lost when it was adjusted for BMI in Model 3. Nevertheless, it is interesting to note that the increase BMI and insulin were not accompanied by significant increases in HOMA_IR_, indicating a lack of association with insulin resistance. The absence of association with waist circumference and percentage of body fat, also indicates that the body fat distribution is not consistent with insulin-resistance state, as revealed by the null association with markers of central obesity. An increase in insulin levels not connected to insulin resistance could result counterintuitive since the hyperinsulinemic state is traditionally viewed as a compensatory response to insulin resistance (28). However, new evidence is challenging this paradigm and postulating that the hyperinsulinemia would be a primary exacerbated response of beta cells to chronic overnutrition further followed by insulin resistance as a protective response by periferic tissues. This possibility is consistent with the fact that our sample comprised young subjects (36 years old, on average) without overt metabolic disturbances who could be going through an early phase of hyperinsulineamia. Whatever the reason for this dilemma, it is also possible that the modest sample size of our study may also have failed to show association with markers of insulin resistance due to the low statistical power. Other study in Mexican population have reported decreased insulin action, together with higher ALT and GGT levels in risk haplotype carriers with T2D (29). Since both studies support the involvement of the risk haplotype in the development of insulin resistance, this genetic variant could represent an early marker of T2D. In agreement with this proposal, the GWAS developed by the SIGMA Consortium, reported that the risk haplotype advances the development of T2D by 2.1 years and that its association with T2D was stronger in younger people (12). Furthermore, a case-control and case-parent trio study found an association between *SLC16A11* and the risk of pediatric-onset T2D in Mexican families (30). The association between the risk haplotype and increased BMI that we report is striking because previous studies have shown an association in the opposite direction but only among diabetics (30). Analysis of longitudinal data suggests that risk haplotype carriers lose more weight than noncarriers do after diabetes onset (31). As our data were obtained from a young nondiabetic population which could represent an early stage in the progression of diabetes, we believe that it is possible that the participants bearing the risk allele may have experienced weight loss after the establishment of T2D. This hypothesis will require further testing in a longitudinal study starting several years before the onset of diabetes.

Over 90% of SNPs associated with obesity are located in noncoding regions or even in intergenic regions of the genome (32). These locations make it difficult to define causal relationships between the SNP and disease-related functional alterations. This is the case for the obesity-susceptibility alleles in the first intron of the *FTO* gene, whose connection to obesity has been linked to their role as a cis-regulatory element of the IRX3 transcription factor (33). In contrast, the SNP studied here directly causes a missense mutation, which initially was reported to be associated with reduced expression and abnormal subcellular localization, causing functional impairment of the monocarboxylate 11 transporter in the liver (14). Recently, Zhao and cols. reported conflicting data showing that *SLC16A11* ablation in the knockout mice did not provoke metabolic alterations related to T2D. Only the reincorporation of the mutated *SLC16A11* gene into the knockout rendered a mouse that developed excessive lipid accumulation and insulin resistance when fed a high fat diet (34). In line with the latest evidence, Zhang and cols. reported that reducing hepatic *SLC16A11* expression prevented triglyceride accumulation in the liver and maintained glucose tolerance in mice fed a high-fat diet (35). Interestingly, expression of the wildtype *SLC16A11* was induced in the liver of mice by a high fat diet and reduced by endurance exercise, which suggests that the gene is deeply involved in the sensing of lifestyle changes (35). In this sense, it is possible that the association between the haplotype of *SLC16A11* and T2D may depend largely on environmental conditions, which would explain why the association with T2D is heterogeneous among Latin American populations with very similar genetic backgrounds but living in diverse environments.

### Limitations:

A limitation of our study was the selection of a population with no history of metabolic diseases and with an average age of under 40 years, which prevents establishing an association of the polymorphism rs75493593 with obesity and T2D. Nonetheless, a positive association between *SLC16A11* risk genotypes with BMI and insulin was found in nondiabetic individuals, which indicates that the SNP can be considered an early risk marker for obesity and T2D. Another limitation of our study was the small sample size, which precluded association analysis for specific groups of the population, such as obese versus normal subjects. However, the statistical power was sufficient to find an association between the haplotype and both increased BMI and insulin. An eQTL analysis conducted to explore whether the association of the SNP rs75493593 are mediated by changes in the expression of the gene product would have been informative, which also constitutes a limitation of our study.

In conclusion, the data presented here shows that the SNP rs75493593 in the *SLC16A11* gene has an allele frequency of 29.7% and is associated with an increased BMI and insulin levels in the Chilean population. It will be important to perform new studies in order to estimate the contribution of different genetic variants to the development of highly prevalent diseases, such as obesity and T2D, in order to facilitate the timely identification of at risk individuals for preventive interventions. Recent mechanistic studies have attempted to elucidate the link between the functional alteration of *SLC16A11* and the pathogenesis of T2D (34–36). This information will pave the way for targeting this solute carrier, for personalized T2D treatment and prevention.
